# Tightly linked *Rps12* and *Rps13* genes provide broad-spectrum *Phytophthora* resistance in soybean

**DOI:** 10.1038/s41598-021-96425-1

**Published:** 2021-08-19

**Authors:** Dipak K. Sahoo, Anindya Das, Xiaoqiu Huang, Silvia Cianzio, Madan K. Bhattacharyya

**Affiliations:** 1 Department of Agronomy, Iowa State University, Ames, IA 50011 USA; 2 Department of Computer Science, Iowa State University, Ames, IA 50011 USA

**Keywords:** Plant sciences, Plant breeding, Plant genetics

## Abstract

The *Phytophtora* root and stem rot is a serious disease in soybean. It is caused by the oomycete pathogen *Phytophthora sojae*. Growing *Phytophthora* resistant cultivars is the major method of controlling this disease. Resistance is race- or gene-specific; a single gene confers immunity against only a subset of the *P. sojae* isolates. Unfortunately, rapid evolution of new *Phytophthora sojae* virulent pathotypes limits the effectiveness of an *Rps* (“resistance to *Phytophthora sojae*”) gene to 8–15 years. The current study was designed to investigate the effectiveness of *Rps12* against a set of *P. sojae* isolates using recombinant inbred lines (RILs) that contain recombination break points in the *Rps12* region. Our study revealed a unique *Rps* gene linked to the *Rps12* locus. We named this novel gene as *Rps13* that confers resistance against *P. sojae* isolate V13, which is virulent to recombinants that contains *Rps12* but lack *Rps13*. The genetic distance between the two *Rps* genes is 4 cM. Our study revealed that two tightly linked functional *Rps* genes with distinct race-specificity provide broad-spectrum resistance in soybean. We report here the molecular markers for incorporating the broad-spectrum *Phytophthora* resistance conferred by the two *Rps* genes in commercial soybean cultivars.

## Introduction

Soybean [*Glycine max* (L.) Merr.] is one of the main oilseed crops produced and consumed worldwide and is among the world’s five utmost significant food crops^1^. Its production is limited by several soybean diseases, with an average annual yield loss of 11% in the United States^2^. *Phytophthora* root and stem and root rot (PRS) disease is in the top five diseases that cause severe annual yield losses in soybean^3^. The annual crop damage from PSR between 2003 and 2005 averaged about $251.6 million^3,4^. From 2010 to 2014, in 28 US states and Ontario, Canada, PSR caused an estimated annual yield loss of $403 millions^3^. The PSR disease in soybean is caused by the soil-borne oomycete *Phytophthora sojae*^5,6^, and soybean plants infected with *P. sojae* are more susceptible to infection by other soil-borne pathogens.

Oomycete pathogens are challenging to control and most fungicides are ineffective because the *P. sojae* infected roots are difficult to treat effectively with chemicals. Another difficulty is that many oomycetes appear to have extraordinary genetic flexibility, enabling them to adapt to and overcome rapidly the chemical control measures as well as host resistance mechanisms^7,8^. Methods employed to control PRS include fungicide applications^9^, planting resistant cultivars^10,11^, improvement in soil drainage^8^, modification of tillage practices^12^, and application of calcium-containing compounds^13,14^. The most effective way to reduce PRS damage is planting *Phytophthora* resistant soybean cultivars^11^.

Single dominant *Rps* genes confer resistance against *P. sojae* isolates that carry the cognate avirulence (*Avr*) gene. Soybean *Rps* genes activate effector-triggered immune responses^15^, as in other pathosystems^16^. More than 30 *Rps* genes/alleles have been mapped to nine chromosomes, including the newly identified *Rps* genes, *RpsGZ* and *RpsX*^17,18^. The *Rps1* locus contains five functional alleles (*Rps1a, 1b, 1c, 1d,* and *1k*)^19–21^, and the *Rps3* locus contains three (*Rps3a, 3b,* and *3c*)^21,22^. The *Rps* genes mapped to Chromosome 3 include *Rps1, Rps7, Rps9, RpsYu25, RpsYD29, RpsYD25, RpsUN1, Rps1?* and *RpsWY*^14,20,23–28^. While *Rps2* gene and *RpsUN2* have been mapped to Chromosome 16^24,29,30^, and the three *Rps3* alleles, *Rps3a, Rps3b,* and *Rps3c* along with *Rps8* and *RpsSN10* to Chromosome 13^22,24,31–33^. The *Rps4, Rps5, Rps6, Rps12,* and *RpsJS* genes are tightly linked and are located on the lower arm of Chromosome 18^17,24,34–36^. *Rps10* has been mapped to Chromosome 17^23^, *RpsYB30*, and *RpsZS18* to Chromosome 19^33,37^ and Chromosome 2^37^, respectively, and *Rps11* to Chromosome 7^38^.

*P. sojae* isolates evolve rapidly to overcome the introduced resistance genes in commercial cultivars, especially under the monoculture scenario. Over 200 known pathotypes of this pathogen have been reported and the number is ever growing presumably due to selection pressure on the *P. sojae* population for new pathotypes that can overcome the newly introduced *Rps* genes. The rapid evolution of new *P. sojae* virulent pathotypes limits the effectiveness of an *Rps* gene to 8–15 years^39^. For example, a survey on pathotype changes in the population of *P. sojae* over several decades showed that while 6% of the pathotypes could defeat the *Rps1c* gene from 1991 to 1994, it was 57% by 2004. While in 1994, no pathotype could defeat *Rps1k*, the number of pathotypes increased to 12% in 2004, and to 41% in 2015. The number of pathotypes that defeat both *Rps1c* and *Rps1k* increased from none to 31% between 1994 and 2015^39^. With increased complexity of *P. sojae* pathotypes, new strategies for managing this pathogen are needed^39^. The use of resistant cultivars is the most cost-effective and environmentally safe method to control this disease. Henceforth, there is a constant need for novel *Rps* (“resistance to *Phytophthora sojae*”) genes to manage the disease effectively.

It was suggested that plant introduction (PI) line, PI399036 contains multiple *Rps* genes^40,41^. An *Rps* gene, *Rps12*, from this PI line was mapped to a 5.4 cM region between the simple sequence repeat (SSR) marker BARCSOYSSR_18_1840 and the NBSRps4/6-130/533 sequence^17^. To determine the utility of *Rps12,* we investigated the responses of recombinant inbred lines (RILs) containing *Rps12* against a collection of *P. sojae* isolates. The objective of this study was to investigate the effectiveness of *Rps12* against different *P. sojae* isolates. We utilized a set of recombinant inbred lines (RILs) containing recombination break points in the *Rps12* region and lacking functional *Rps* genes in other known *Rps* loci. Investigation of the selected set of RILs for responses to a set of *P. sojae* isolates collected in Iowa revealed *Rps13*, linked tightly to *Rps12* on Chromosome 18. The *Rps12* and *Rps13* genes together provide broad-spectrum *Phytophthora* resistance in soybean.

## Materials and methods

### Plant genetic material

The AX20925 RIL population used in this study was developed by crossing PI399036 with the germplasm line AR2. This population was used earlier to map *Rps12*^17^. The individual F_2_ plants were advanced to the F_8_ generation by applying the single-seed descent method^41^. In this study, 120 F_8_ families (recombinant inbred lines, RILs) were phenotyped for responses to a *P. sojae* isolate V13 that overcomes *Rps12* encoded resistance and a mixture of isolates (R17 & Val12-11) that defeat *Rps1a, 1b, 1c, 1d, 1k, 2, 3a, 3b, 3c, 4, 5, 6,* and *7* genes. Of these 120 RILs, 60 were homozygous resistant, and 60 were homozygous susceptible. The 120 RILs were used in molecular mapping of the *Rps13* gene.

### *Phytophthora sojae* isolates

*Phytophthora sojae* isolates R17 (vir 1b, 1d, 3a, 3b, 3c, 5, 6), Val 12-11 (vir 1a, 1b, 1c, 1d, 1k, 2, 4, 7), 1005-2.9 (vir 1a, 1b, 1c, 1k, 3b, 7), III 5.2b (vir 1a, 1b, 1c, 1d, 1k, 7), III 23.4b (vir 1a, 1c, 1d, 2, 3b, 3c, 4, 5, 7), IV 5.2 (vir 1a, 1c, 1d, 2, 7), IV 10 (vir 1a, 1c, 1d, 7), IV 12.2a (vir 2, 4, 7), IV 13.4a (vir 7), IV 23.3 (vir 1a, 1c, 1d, 2, 7), VI 5.2b (vir 1a, 1c, 1d, 7), VI 12.1a (vir 6,7), VI 12.2b (vir 1d, 3a, 4, 5, 6, 7), VI 15 (vir 1d, 7), VI 20 (vir 1c, 2, 7), VI 23.3b (vir 1d, 7), PR1 (vir 7), PR6 (vir 7), S 5-5 (vir 7), V 13 (vir 1a, 1c, 1d, 4,7) and IV 6b (vir 1a, 1c, 1d, 7) were used for investigating the efficacy of *Rps12* (Table 1). The *P. sojae* isolates were obtained from Anne Dorrance (Ohio State University, OH), Martin Chilvers (Michigan State University, MI), and Alison E. Robertson (Iowa State University). All isolates were grown on half-strength V8 agar plates amended with neomycin sulfate and chloramphenicol antibiotics for 5–7 days under room temperature in the dark as described by Dorrance et al. (2008)^42^.

**Table 1:** Response of differential soybean lines and PI 399073 carrying *Rps12* and *Rps13* genes to 21 *Phytophthora sojae* isolates.

Differential line	*Rps* gene	*P. sojae* isolates																				
		III 5.2b	III 23.4b	IV 5.2	IV 6b	IV 10	IV 12.2 a	IV 13.4a	IV 23.3	V 13	VI 5.2b	VI 12.1 a	VI 12.2b	VI 15	VI 20	VI 17	VI 23.3b	S 5-5	R17	Val 12-11	1005-2.9	PR1
L88-8470	*1a*	S	S	R	S	S	R	R	S	S	S	R	R	R	R	R	R	R	R	S	S	R
L77-1863	*1b*	S	R	R	R	R	R	R	R	R	R	R	R	R	R	R	R	R	S	S	S	R
Williams 79	*1c*	S	S	S	S	S	R	R	S	S	S	R	R	R	S	R	R	R	R	S	S	R
L93-3312	*1d*	S	S	S	S	S	R	R	S	S	S	R	S	S	R	R	S	R	S	S	R	R
Williams 82	*1k*	S	R	R	R	R	R	R	R	R	R	R	R	R	R	R	R	R	R	S	S	R
L82-1449	*2*	R	S	R	R	R	S	R	S	R	R	R	R	R	S	R	R	R	R	S	R	R
L83-570	*3a*	R	R	R	R	R	R	R	R	R	R	R	S	R	R	R	R	R	S	R	R	R
L91-8347	*3b*	R	S	R	R	R	R	R	R	R	R	R	R	R	R	R	R	R	S	R	S	R
L92-7857	*3c*	R	S	R	R	R	R	R	R	R	R	R	R	R	R	R	R	R	S	R	R	R
L85-2352	*4*	R	S	R	R	R	S	R	R	S	R	R	S	R	R	R	R	R	R	S	R	R
L85-3059	*5*	R	S	R	R	R	R	R	R	R	R	R	S	R	R	R	R	R	S	R	R	R
L89-1581	*6*	R	R	R	R	R	R	R	R	R	R	S	S	R	R	R	R	R	S	R	R	R
L93-3258	*7*	S	S	S	S	S	S	S	S	S	S	S	S	S	S	S	S	S	R	S	S	S
PI 399073	*8*	R	R	R	R	R	R	R	R	S	R	R	R	R	S	R	R	R	R	R	R	R

Plants were rated seven days after inoculation as either R (resistant, < 30% seedling death) or S (susceptible, ≥ 70% seedling death).

### Evaluation of genetic materials for *Phytophthora* resistance

Hypocotyls of 7-day-old seedlings of 120 RILs, the parents PI399036 and AR2 along with 14 differential lines carrying *Rps1a, Rps1b, Rps1c, Rps1d, Rps1k, Rps2, Rps3a, Rps3b, Rps3c, Rps4, Rps5, Rps6, Rps7,* and *Rps8* genes and the susceptible cultivar ‘Sloan’ with no known *Rps* genes^17,42–44^ were inoculated using the wounded-hypocotyl inoculation technique^45,46^. The experiment was conducted three times. Plants were rated seven days after inoculation as either R (resistant, < 30% seedling death) or S (susceptible, ≥ 70% seedling death). Inocula were prepared using a modified version of the protocol described by Dorrance et al. (2008)^42^. The macerated R17 and Val 12–11 cultures were mixed in equal proportion to prepare the mixed inoculum^45^ that is virulent to soybean cultivars carrying *Rps* genes mapped to *Rps1* to 7 loci and partially virulent to lines carrying *Rps8*. *P. sojae* strain V13 was also used as a separate inoculum as it is virulent to soybean lines carrying any of *Rps1a, 1c, 1d, 4, 7,* and *12* genes.

### DNA preparation, bulked segregant analysis (BSA)

Before inoculation, one unifoliate leaf from each of 11 random plants of individual RIL was collected, bulked and frozen in liquid nitrogen, and stored at − 80 °C. The genomic DNA was extracted from the bulked leaf samples using the CTAB (cetyl trimethyl-ammonium bromide) method^47^. The identified SSR markers linked to *Rps12* locus^17^ were used to conduct BSA for the *Rps13* region on pooled DNA samples of 10 homozygous resistant RILs (Resistant Bulk) or ten susceptible RILs (Susceptible Bulk)^17,48^. In BSA^48^, a polymorphic molecular marker linked tightly to a target locus shows its allelic segregation either in coupling or repulsion phase linkage with alleles of the target locus. In BSA assays, the markers that are not linked to the target locus show heterozygosity due to recombination of the marker alleles with alleles of the target locus.

### PacBio long-read sequencing and development of sequence-based polymorphic (SBP) molecular markers

A ~ 50 genome equivalents genome sequence of the PI399036 and AR2 was obtained by PacBio long-read sequencing at the DNA Facility, Iowa State University. The bowtie program was run to identify single nucleotide polymorphisms (SNPs) between genomes of the resistant (PI399046) and susceptible (AR2) lines by mapping sequence reads onto the 8 Mbp region spanning the 53–61 Mbp physical locations on Chromosome 18 containing *Rps12* and *Rps13*. Over 26,000 putative SNPs were identified. We used SNPs of the putative *Rps13* region to develop necessary SBP markers according to Sahu et al.^49^ for mapping the *Rps13* gene. Among the identified SNPs, we looked for the ones that are polymorphic for restriction endonucleases. Polymerase chain termination reaction (PCR) amplicons of approximately 200 nucleotides DNA containing variations for restriction endonuclease sites between PI399036 and AR2 were considered as putative SBP markers. Finally, primers for PCR amplification were designed in such a way that one can easily distinguish the haplotype-specific restriction fragment length polymorphisms by separating the restriction enzyme digested PCR products on a 4% (w/v) agarose gel^49^. Seventeen SBP markers were identified for the *Rps12*-*Rps13* region (Table S1).

Simple sequence repeats (SSR) and SBP markers were used to construct a linkage map of the genomic region carrying the putative novel *Rps13* gene. Molecular markers based on previously reported NBSRps4/6 sequence and SSR markers^17^, and newly developed SBP markers were used in mapping the *Rps13* gene (Table S1). SSR markers linked to *RpsJS* were also used in mapping the *Rps13* region^34^. Eleven polymorphic SSR markers, two previously reported NBSRps4/6 molecular markers along with the newly developed five SBP markers were used to map the *Rps13* gene^17^ (Tables S1, S2).

### Screening RILs and parental lines for the presence of known *Rps* genes

Twenty-three SSR markers linked to the *Rps1, 2, 3, 7, 8, 9, 10, 11, Yu25, WY, Rps1?, RpsUN1, UN2,* and *YD29* loci were used to evaluate for possible polymorphisms between the AR2 (susceptible), and PI399036 (resistant) parents (Table 2) in order to identify RILs that carry SSR alleles specific to the *P. sojae* susceptible AR2 parent.

**Table 2:** SSR markers linked to known *Rps* regions.

*Rps* gene	Linked SSR markers	Chromosome	Molecular linkage group
*Rps1a, b, c, d, k*	Satt152, Sat_186, Satt631, Satt683, Satt159, Satt530 & Satt009	3	N (Gordon et al.^40^; Sugimoto et al.^14^; Wu et al.^28^; Sun et al.^27^; Lin et al.^29^)
*Rps2*	Sat_144 & Satt440	16	J (Gordon et al.^40^)
*Rps3a, b, c*	Satt335 & Satt510	13	F (Gordon et al.^40^)
*Rps4*	Sat_064	18	G (Sandhu et al.^35^; Sahoo et al.^17^)
*Rps6*	Sat_064	18	G (Sandhu et al.^35^; Sahoo et al.^17^)
*Rps7*	Satt631, Satt683, Satt152, Satt530 & Satt009	3	N (Gordon et al.^40^; Sugimoto et al.^14^; Sun et al.^27^)
*Rps8*	Satt663	13	F (Gordon et al.^40^)
*Rps9*	Satt631 & Sat186	3	N (Wu et al.^28^; Lin et al.^29^)
*Rps10*	Sattwd15-24, Sattwd15-25 & Sattwd15-47	17	D2 (Zhang et al.^23^)
*Rps11*	SSR_07_0286, SSR_07_0300 & SSR_07_0295	7	M (Ping et al.^38^)
*Rps12*	BARCSOYSSR_18_1840 & Sat_064	18	G (Sahoo et al.^17^)
*UN1*	Satt159 & SSR_03_0250	3	N (Lin et al.^29^)
*RpsUN2*	SSR_16_1275 & Sat_144	16	J (Lin et al.^29^)
*RpsYu25*	Sat186 & Satt152	3	N (Sun et al.^27^; Lin et al.^29^)
*YD29*	SattWM82-50 and Satt1k4b	3	N (Zhang et al.^23^)
*RpsWY*	Satt631 & Satt152	3	N (Cheng et al.^75^)
*Rps1?*	Satt631, Sat186 & Satt009	3	N (Sugimoto et al.^14^)
*RpsJS*	SSRG60684K & BARCSOYSSR_18_1861	18	G (Sun et al.^14^)

### Linkage map construction and statistical analysis

The Chi-square (χ^2^) analysis was performed to check the phenotypic data for goodness-of-fit to a Mendelian segregation 1:1 ratio using Graphpad (http://www.graphpad.com/quickcalcs). Mapmaker version 3.0^50^ and the Kosambi mapping function^51^ were used to calculate genetic distances in cM units from the recombination fractions between any given two loci. A logarithm of the odds (LOD) threshold was set as 3.0 to determine the linkages between studied loci. Mapmaker package uses the Lander-Green algorithm to calculate the “best” map order of loci^50^. The marker order was determined using the log-likelihood method^50^. The linkage map of molecular markers and the *Rps* genes was drawn using MapChart 2.3^52^.

### The source of *Rps12* and *Rpas13* genes

The PI399036 containing the two *Rps* genes, *Rps12* and *Rps13,* is available from the USDA Soybean Germplasm Collection. The contact person for the seeds is Esther K Peregrine (esther.peregrine@ars.usda.gov), Assistant Soybean Curator, USDA/ARS SoybeanSoybean Germplasm Collection, 1101W. Peabody Dr., Rm. 180, National Soybean Research Center, Urbana, IL 61801, USA.

The segregating materials studied in this study were generated by author Silvia Cianzio and will be available from the Bhattacharyya lab, G319 Agronomy Hall, Iowa State University, Ames, IA 50011, USA. All plant collection methods were complied with relevant institutional, national, and international guidelines and legislation.

## Results

### Identification of putative RILs carrying the *Rps12* gene

It was proposed that the *Phytophthora* resistant PI399036 line contains multiple *Rps* genes^40^. Earlier we mapped *Rps12* of this line using a mixture isolates that overcome most known *Rps* genes^17^. To investigate the utility of *Rps12* against a set of *P. sojae* isolates collected from Iowa soybean field, we looked for RILs that carry only *Rps12*. We have investigated 60 *Phytophthora* resistant RILs generated from the cross between PI399036 × AR2^17^ for SSR markers linked to the known *Rps* regions as described below.

**Figure 1:**
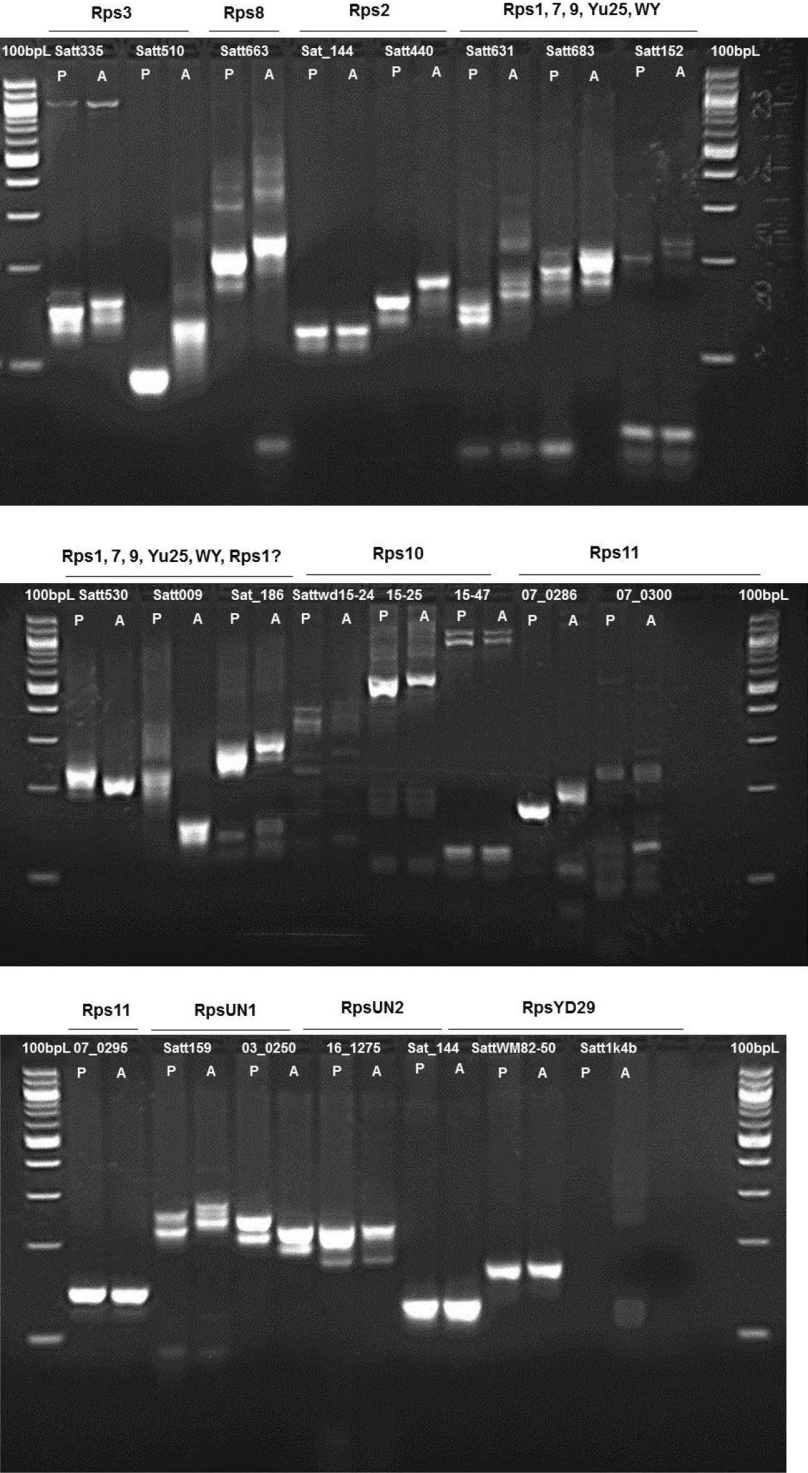
Polymorphisms between resistant (P) and susceptible (A) parents for the SSR markers linked to different *Rps* regions. *P* resistant parent PI399036; *A* susceptible parent AR2.

We used 23 SSR markers that were published earlier (Table 2). These include SSR markers Satt335 and Satt510 for *Rps3* locus, Satt663 for *Rps8* locus, Sat_144 and Satt440 for *Rps2* locus, and Satt631, Satt683, Satt152, Satt530, Satt009 and Sat186 for *Rps1, 7, 9, Yu25, WY,* and *Rps1?* Loci, Sattwd15-24, Sattwd15-25 and Sattwd15-47 for *Rps10*, SSR_07_0286, SSR_07_0300 for *Rps11* and SSR_07_0295 for *Rps1*, Satt159 and SSR_03_0250 for *RpsUN1*, SSR_16_1275 and Sat144 for *RpsUN2*, and SattWM82-50 and Satt1k4b for *RpsUD29* locus^14,27,38,40^ (Table 2). The 23 SSR markers were investigated for polymorphisms between the resistant PI399036 and susceptible AR2 parents. Of the 23 SSR markers, 10 SSR markers were polymorphic between the two parents (Fig. 1) and applied initially in evaluating all 60 RILs homozygous for *Rps12*; and subsequently, 60 *Phytophthora* susceptible RILs (*rps12rps12*). The ten polymorphic SSR markers considered for this study include Satt510 for *Rps3* locus, Satt663 for *Rps8*, Satt440 for *Rps2* and Satt631, Satt152 and Satt009 for *Rps1, 7, 9, Yu25, WY,* and *Rps1?*, Sattwd15-24 for *Rps10*, SSR_07_0286 for *Rps11*, and Satt159 and SSR_03_0250 for *RpsUN1* (Table S3). From screening of the 60 resistant RILs, we identified RILs 12 and 14 that carry AR2-specific SSR alleles for nine and eight SSR markers, respectively. For RIL12, SSR marker linked to *Rps11* is heterozygous; and for RIL14, two SSR markers linked to *Rps8* and *Rps11* are heterozygous. These two lines were selected to determine the efficacy of *Rps12* to a set of *P. sojae* isolates.

### Identification of *Rps13*

We have obtained 17 *P. sojae* isolates from the Robertson lab, Iowa State University, collected earlier from the Iowa soybean fields. The isolates were characterized for their pathotypes by inoculating a set of 14 soybean lines that are considered to be differential lines for 14 individual *Rps* genes (Table 1). All these isolates were used to infect the differential cultivars and selected RIL12 and RIL14 and two parents, PI399036 and AR2. RIL12 and RIL14 contain *Rps12* and confers resistance against the isolate mixture of R17 and Val 12-11^17^. Surprisingly, RIL12 is not resistant against seven of the 17 new *P. sojae* isolates and Val 12-11 (Table 1). On the contrary, RIL14 is resistant against these seven isolates. Based on the genetic make-ups of RIL12 and RIL14 for molecular markers of the *Rps12* region, we deducted that there is recombination breakpoint in between the NBSLRR533 and Sat_064 in RIL12. We hypothesized that there could be a novel *Rps* gene named *Rps13* located in between *Rps12* and telomere on Chromosome 18.

To further support our hypothesis that there is an *Rps* gene next to *Rps12*, we evaluated 60 *Phytopthora* resistant RILs (*Rps12Rps12*) and 60 *Phytopthora* susceptible RILs (*rps12rps12*) for molecular markers of the genomic region containing the *Rps12* gene (Table S4). From molecular mapping of the 120 RILs, we were able to identify two additional RILs, RIL9 and RIL81, that carry recombination breakpoints in the *Rps12* region, and were evaluated for their responses to 17 new *P. sojae* isolates, and mixture of R17 and Val 12-11 isolates. The RIL12 contains *Rps12,* but not the putative *Rps13* region; whereas, RIL81 contains the putative *Rps13* region but not *Rps12*. RIL9 contains the putative *Rps13* region, but not *Rps12*. RILs that carry *Rps12,* but not *Rps13,* were susceptible to the *P. sojae* isolates, V13, IV 6b and Val 12-11, resistant to R17 (Table 3). On the contrary, RIL81 carrying *Rps13* but not *Rps12* was susceptible to *P. sojae* isolate R17 (Table 3). Our results established that *Rps12* is overcome by several *P. sojae* isolates, against which *Rps13* provides immunity.

**Table 3:** Response of four RILS and their parents to 23 *P. sojae* isolates along and a few *P. sojae* isolate mixtures.

*P. sojae* isolate	Sloan	PI399036 (*Rps12, Rps13*)	AR2 (*rps12, rps13*)	RIL12 (*Rps12, rps13*)	RIL81 (*rps12, Rps13*)	RIL14 (*Rps12, Rps13*)	RIL9 (*Rps12, rps13*)
R17 (vir 1b, 1d, 3a, 3b, 3c, 5, 6)	S	R	S	R	S	R	R
Val 12-11 (vir 1a, 1b, 1c, 1d, 1k, 2, 4, 7)	S	R	S	R	R	R	R
1005-2.9 (vir 1a, 1b, 1c, 1k, 3b, 7)	S	R	S	R	R	R	R
III 5.2b (vir 1a, 1b, 1c, 1d, 1k, 7)	S	R	S	R	R	R	R
III 23.4b (vir 1a, 1c, 1d, 2, 3b, 3c, 4, 5, 7)	S	R	S	R	R	R	S
IV 5.2 (vir 1c, 1d, 7)	S	R	R	R	R	R	R
IV 6b (vir 1a, 1c, 1d, 7)	S	R	S	S	R	R	S
IV 10 (vir 1a, 1c, 1d, 7)	S	R	S	R	R	R	R
IV 12.2a (vir 2, 4, 7)	S	R	S	R	R	R	R
IV 13.4a (vir 7)	S	R	S	R	R	R	R
IV 23.3 (vir 1a, 1c, 1d, 2, 7)	S	R	S	R	R	R	R
V 13 (vir 1a, 1c, 1d, 4, 7)	S	R	S	S	R	R	S
VI 5.2b (vir 1a, 1c, 1d, 7)	S	R	S	R	R	R	R
VI 12.1a (vir 6,7)	S	R	S	R	R	R	R
VI 12.2b (vir 1d, 3a, 4, 5, 6, 7)	S	R	S	S	S	R	S
VI 15(vir 1d, 7)	S	R	S	R	R	R	R
VI 17 (vir 7)	S	R	S	R	R	R	R
VI 20 (vir 1c, 2, 7)	S	R	S	S	R	R	S
VI 23.3b (vir 1d, 7)	S	R	S	R	R	R	R
S 5-5 (vir 7)	S	R	S	R	R	R	R
P7074 (vir 1b, 1d, 2, 3a, 3b, 3c, 4, 5, 6, 7, 8)	S	R	S	R	S	R	R
PR1 (vir 7)	S	R	S	R	R	R	R
PR6 (vir 7)	S	R	S	R	R	R	R
III5.2b + R17 + V13 (vir 1a, 1b, 1c, 1d, 1k, 3b, 5,7,8)	S	R	S	S	S	S	S
1005–2.9 + VI23.3b + R17 (vir 1d, 2, 3b, 7)	S	R	S	R	R	R	R
R17 + Val 12–11 (vir 1a, 1b, 1c, 1d, 1k, 2, 3a, 3b, 3c, 4, 5, 6, 7)	S	R	S	R	S	R	R

Plants were rated seven days after inoculation as either R (resistant, < 30% seedling death) or S (susceptible, ≥ 70% seedling death).

### Molecular mapping of the *Rps13* gene

**Figure 2:**
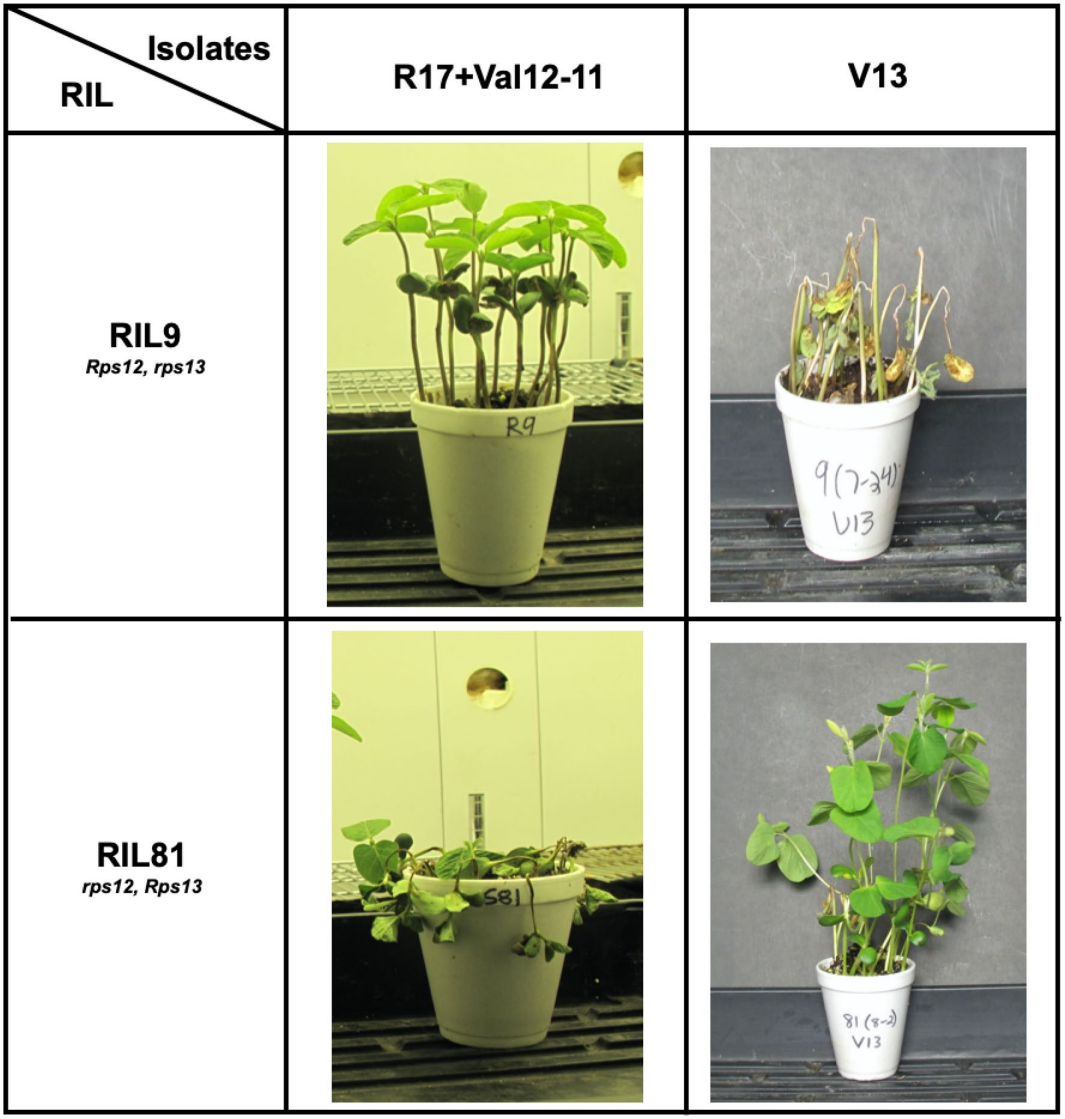
Two RILs differing alleles at the linked *Rps12* and *Rps13* loci showed distinct responses to *P. sojae* V13 isolate and the mixture of R17 and Val12-11 isolates. *P. sojae* isolate V13 failed to defeat resistance mediated by *Rps13* gene but could overcome that by *Rps12*; whereas, the mixture of R17 and Val12-11 isolates could overcome *Rps13*, but not *Rps12*.

**Figure 3:**
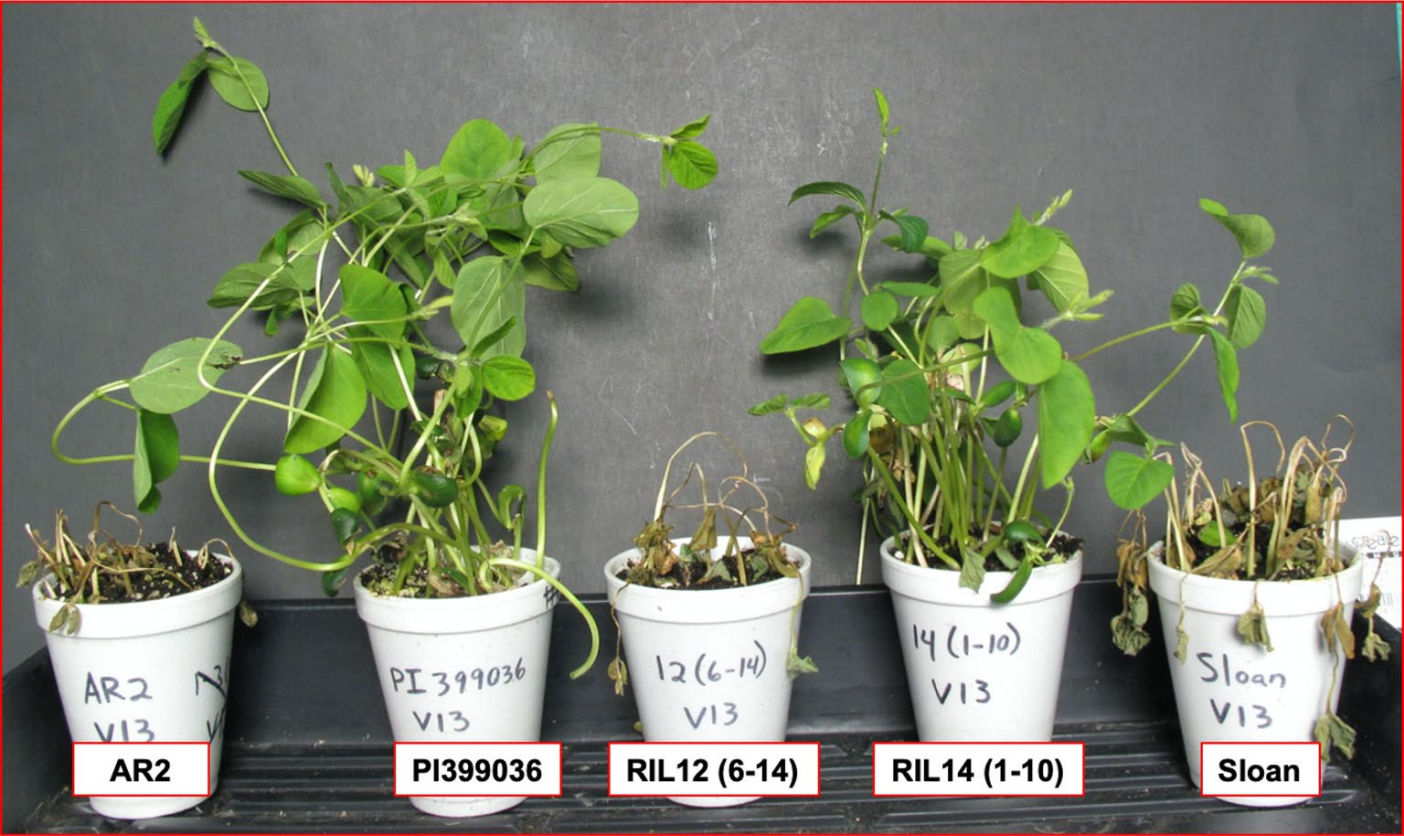
*Phytophthora sojae* isolate V13-specific resistance is conferred by *Rps13.* Reactions of susceptible parent AR2, resistant parent PI399036 containing *Rps12* and *Rps13* genes, recombinant inbred line RIL12 (6–14) susceptible to *P. sojae* isolate V13 due to absence of *Rps13*, recombinant inbred line RIL14 (1–10) resistant to *P. sojae* isolate V13 due to presence of *Rps13* and the susceptible cultivar Sloan with no known *Rps* genes is susceptible to V13 isolate.

We determined the inheritance of the putative novel *Rps13* gene by evaluating 120 RILs for segregation of *Phytophthora* resistance against an inoculum mixture of Val 12-11 and R17, which together are virulent on soybean lines carrying all *Phytophthora* resistance genes mapped to the *Rps1* to *7* loci and partially virulent to lines carrying *Rps8* along with V13 isolate which is virulent to soybean lines carrying *Rps1a, 1c, 1d, 4, 7* and *Rps12* (Figs. 2, 3, Table 3).

Analysis of *Rps* gene-linked SSR markers revealed that alleles of Satt009 and Satt510 markers specific to *Rps1c* and *Rps3a* alleles, respectively, are present in PI399036, but not in AR2. We hypothesized that most likely PI399036 contains *Rps1c* and *Rps3a*, in addition to *Rps12* and *Rps13*. *P. sojae* isolate V13 overcomes the resistance conferred by *Rps1c*, but not *Rps3a*. We therefore classified the RILs into two groups based on Satt510: (i) The RILs which carry Satt510 allele specific to *rps3a* and AR2 parent; and (ii) RILs carry Satt510 allele specific to *Rps3a*. Both groups segregated for resistance to susceptibility in a 3:1 ratio, as observed for single Mendelian genes, following infection with *P. sojae* V13 isolate that overcomes the resistance governed by *Rps12* and *Rps1c.* This confirms that there is a novel *Rps* gene in PI399036.

To map the novel gene, the 120 RILs from the AX20925 population were infected with a mixture of *P. sojae* R17 and V13 isolates that together overcome all known *Rps* genes including *Rps12*, but not the novel *Rps13* gene. Of the 120 RILs, 52 RILs showed resistance against the isolate mix and 67 showed susceptibility. The observed segregating 0.867:0:0.08:1.117 genotypic ratio of resistance to susceptibility among the 120 RILs fits to the expected 0.984:0.032:0.984::RR:Rr:rr ratio, where R is *Rps13* and r is *rps13* for single gene segregation among the RILs in F_7_ generation with an estimated 98.4% of the genes homozygous (χ^2^ = 0.104).

**Figure 4:**
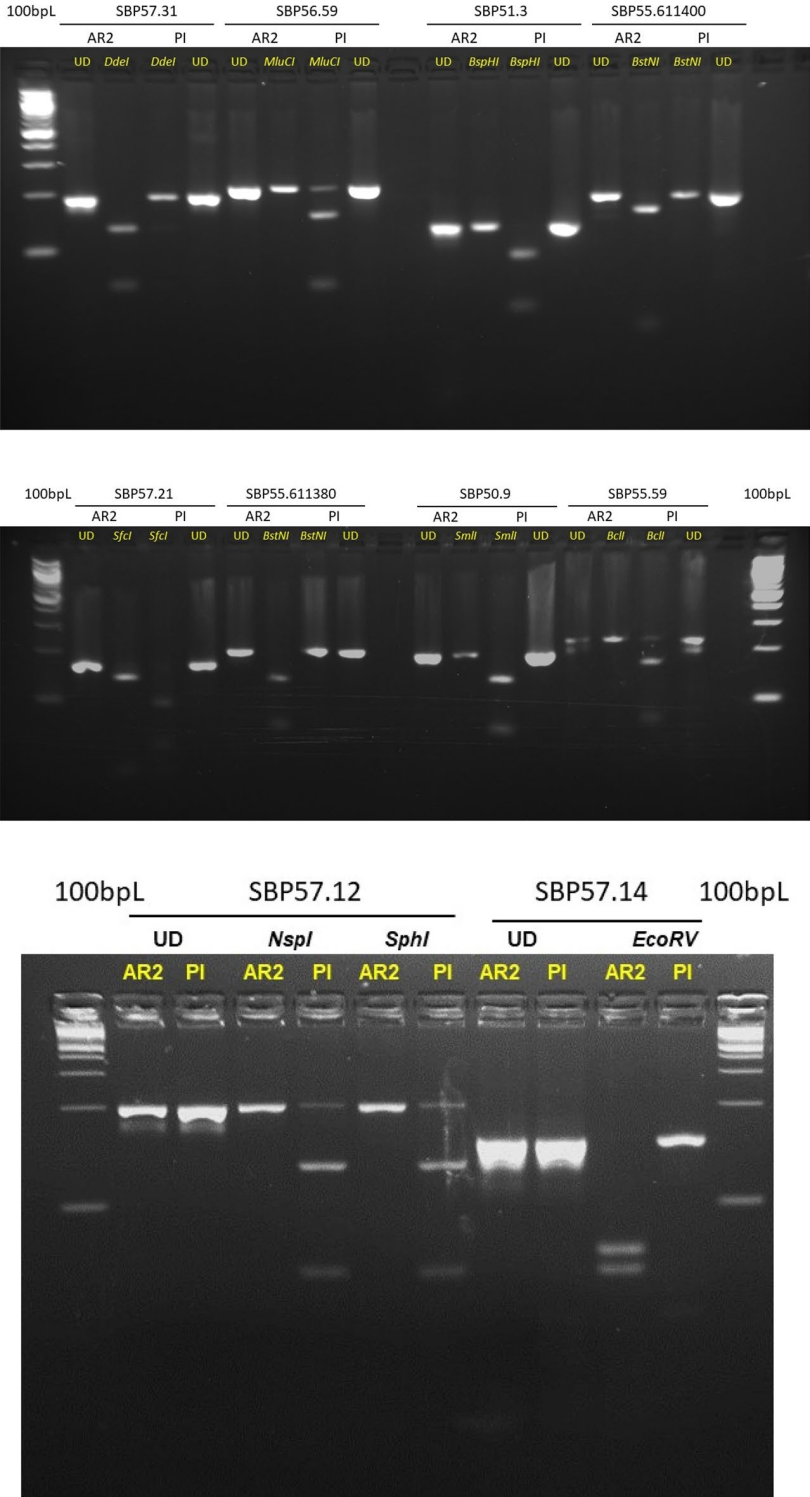
Identification of sequence-based polymorphic (SBP) markers linked to *Rps12* and *Rps13* genes. *AR2* susceptible parent AR2; *PI* resistant parent PI399036; Undigested (UD) and digested (as marked with respective restriction enzymes) PCR products for the SBP markers: SBP57.31, SBP56.59, SBP51.3, SBP55.611400, SBP57.21, SBP55.611380, SBP50.9, and SBP55.59. Primers and enzymes used for SBP markers are presented in Table S1.

**Figure 5:**
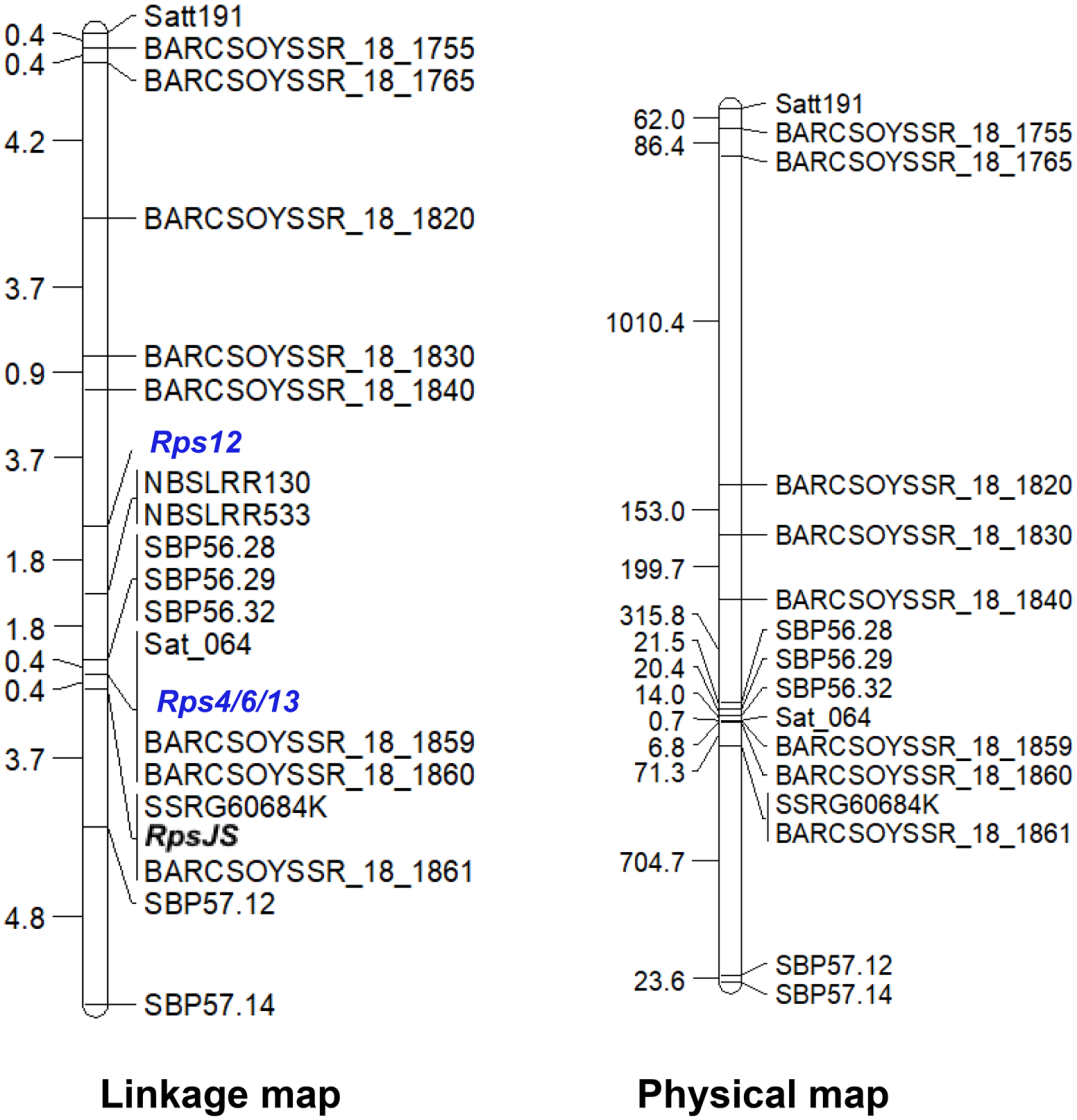
Linkage and physical map of the *Rps4/6/12*/*13/JS* region. (**A**) Genetic map of the *Rps12*-*Rps13-RpsJS* region. SSR and SBP markers are shown on the right side of the map and corresponding genetic distances between two adjacent loci in centi-Morgan (cM) on the left side of the map. *Rps13* gene is mapped between *Rps12* and *RpsJS* and tightly linked to Sat_064, BARCSOYSSR_18_1859 and BARCSOYSSR_18_1860 SSR markers. The placement of *Rps4*, *6*, and *JS* on the map is based published work^23,38^. (**B**) The physical map positions of the SSR and SBP markers are based on the cultivar ‘Williams 82’ genome sequence (http://soybase.org). The physical distances between adjacent loci are presented in kilobases DNA (shown on the left side of the map).

We conducted bulked segregant analysis (BSA) to identify molecular markers linked to the novel *Rps13* resistance gene and confirm that *Rps13* is mapped next to *Rps12*^48^. In this BSA study, we used SSR markers of the *Rps12* region to test our hypothesis that *Rps13* is linked to *Rps12*. The results of BSA suggested that indeed the gene is co-segregated with the markers mapped in between *Rps12* and telomere. To develop a high-resolution map of the *Rps13* region, we investigated 19 putative SBP markers for polymorphisms. Five of the 19 putative SBP markers are polymorphic between resistant and susceptible parents and were used for mapping the *Rps12*-*Rps13* region (Fig. 4). The *Rps13* gene co-segregated with the Sat_064, BARCOSOYSSR_18_1859, and BARCOSOYSSR_18_1860 markers. The genetic distance between *Rps12* and *Rps13* genes is 4 cM (Fig. 5, Table S4).

To identify homologues of the candidate *Rps13* genes*,* we investigated the annotated soybean genes in the 92.7 kb *Rps13* region between the two markers, SBP56.32 and BARCSOYSSR_18_1861, in the Williams 82 genome sequence located at the soybean genome browser (SoyBase; https://www.soybase.org)^53^. Sixteen genes including an NB-ARC domain-containing disease resistance-like gene, *Glyma.18g283200*, are present in this region (Table S5). Williams 82 does not carry the *Rps13* gene.

## Discussion

This study was designed to investigate the usefulness of the *Phytophthora* resistance governed by the *Rps12* gene^17^. In mapping *Rps12*, we had to use a mixture of *P. sojae* isolates to mask the effect of previously known *Rps* genes that were in the PI399036 line, the source of *Rps12*^17^. To determine the utility of *Rps12* genes against a set of uncharacterized *P. sojae* isolates, we must identify an RIL that contains only *Rps12*. We therefor first examined a set of 60 *Phytophthora* resistant RILs for possible absence of other known *Rps* genes by studying the polymorphisms of *Rps* gene-linked SSR markers. Linked SSR markers co-evolved with linked *Rps* genes and SSR alleles can be used to predict alleles of the linked *Rps* genes.

A total of 210,990 SSRs were identified from the soybean genome. Of these, 61,458 SSRs contain repeat units of di-, tri-, and tetranucleotide with (AT)n, (ATT)n and (AAAT)n as the most abundant motifs^54^. A genetic linkage map consisting of 20 linkage groups with approximately 1500 SNP, 1000 SSR markers, 700 RFLP, and 73 RAPD markers and 46 classical trait loci is available in soybean^55–57^. Information of genetic markers has been used to map *Rps1, Rps2, Rps3, Rps4, Rps5, Rps6, Rps7,* and *Rps8* loci to Chromosomes 3, 16, 13, 18 and 13, respectively^24,25,31,32,35,57–59^. While the *Rps4* locus was mapped close to the *Rps6* region, the *Rps8* locus mapped close to the *Rps3* region^31,32,35^. The RFLP marker pT-5 was shown to be linked to the *Rps5* locus^36^. SSR markers mapped to the *Rps5* locus are yet to be identified^24^. Thus, SSR markers linked to each *Rps* gene except *Rps5* have been reported^24^.

In this study, we selected 23 SSR markers that have been shown to be linked to most of the reported *Rps* genes (Table 2). Out of 21 SSR markers, 10 were polymorphic between the two parents, PI399036 and AR2 (Fig. 1). These 10 SSR markers were applied in evaluating all 60 RILs homozygous for *Rps12*. These polymorphic markers included Satt510 linked to the *Rps3* locus, Satt663 to *Rps8*, Satt440 to *Rps2*, and Satt631, Satt152 and Satt009 to *Rps1, 7, 9, Yu25, WY, Rps1?*, Sattwd15-24 to *Rps10*, SSR_07_0286 to *Rps11*, Satt159 and SSR_03_0250 to *RpsUN1*. PI399036, the source of *Rps12*, exhibitted the alleles of the Satt009 and Satt510 linked to linked to *Rps1c* and *Rps3a* alleles, respectively, suggesting that PI399036 most likely contains *Rps1c* and *Rps3a* genes as well.

From PCR assays of 60 *Phytophthora* resistant RILs with 10 SSR markers polymorphic between PI399036 and AR2, we identified RIL12 and RIL14 carrying *rps* alleles-specific SSR alleles for nine and eight SSR markers, respectively. For RIL12, SSR marker linked to *Rps11* is heterozygous and for RIL14, two SSR markers linked to *Rps8* and *Rps11* are heterozygous. These two lines were selected to determine the efficacy of *Rps12* to a set of 17 *P. sojae* isolates collected in Iowa soybean fields. RIL12 was susceptible to seven of the 17 *P. sojae* isolates; whereas, RIL14 was resistant to these seven isolates. Earlier both lines were shown to carry *Rps12*^17^. We hypothesize that RIL12 lacks an unknown *Rps* gene that is present in RIL14. In the absence of this unknown gene the RIL12 failed to provide immunity against four of the 17 isolates studied (Table 3). The putative unknown gene is named as *Rps13*. BSA revealed that the gene is linked to *Rps12.* Genetic mapping using 18 molecular markers placed the gene on the south arm of Chromosome 18, at a 4 cM genetic distance from *Rps12.* We observed that due to the absence of *Rps13* in RILs 6, 9, 42, and 49 resulted in susceptibility to the *P. sojae* isolate V13. However, the four lines contain *Rps12* and resistant to the mixture of the isolates, R17 and Val 12-11 that cannot overcome resistance encoded by *Rps12*. On the contrary, RIL81 contains *Rps13* but not the *Rps12* gene. Therefore, this RIL is resistant to V13 and susceptible to the mixture of R17 and Val 12-11 isolates (Fig. 2). These results established that there is a novel gene next to *Rps12* that is essential for immunity of the RILs against four of the 17 *P. sojae* isolates collected in Iowa. Two linked functional *Rps* genes provide broad-spectrum resistance against *P. sojae* isolates tested in this study.

Plant activates defenses against pathogen attacks, determined by a corresponding pair of genes, a gene for avirulence in the pathogen and a gene for resistance (*R*) in the host. Such resistance mechanisms function in both major classes of flowering plants, dicots, and monocots. Clustering of *R* genes at a single locus is a well-reported, and many *R* genes are clustered in plant genomes, including soybean^60^, common bean^61^, *Arabidopsis*^62–64^, *Brassicaceae*^62^, wild potato^65^, tomato^66,67^, coffee trees^68^, wheat^69^ and rice^70,71^. The clustered distribution of *R*-genes provides a reservoir of genetic variation from which new pathogen specificity can evolve through gene duplication, ectopic recombination, unequal crossing-over and diversifying selection^72^. These clusters frequently comprise tandem arrays of genes that regulate resistance to multiple pathogens and to multiple variants of a single pathogen. The clusters may be tight with a little intervening sequence as 20 kb between two functional *Rps1*-k genes in soybean^26,73^, the *RPP5* cluster in *Arabidopsis thaliana* spans 91 kb^64^, or be spread over several megabases as the *Resistance Gene Candidate2* (*RGC2*) locus in lettuce (*Lactuca sativa*)^74^. In rice, also Chromosome 11 is highly enriched in *R*-genes, mostly in clusters; up to 201 loci encode the domains of NBS-LRR and LRR—receptor-like kinase (LRR-RLK) or wall-associated serine/threonine protein kinase (WAK)^70^.

The *Rps12*-*Rps13* region is rich in *Rps* genes. As of now, *Rps4*, *6, 12, 13* and *JS* are mapped to the same genomic region spanning probably less than 5 cM in different soybean haplotypes (^17,34,35^ this work). Earlier we demonstrated that *Rps4* and *Rps6* are allelic and *Rps4* co-segregates with Sat_064^35^. Therefore, most likely *Rps13* is allele to *Rps4* and *Rps6*. The PI399036, the donor of *Rps12* and *Rps13*, does not carry *Rps4* or *Rps6* and therefore *Rps13* is distinct from the two *Rps* genes^17^, this study.

The *Rps13* locus is very close to the *RpsJS* locus (Fig. 5). *Rps13* co-segregates with Sat_064, BARCSOYSSR_18_1859 and BARCSOYSSR_18_1860, and *RpsJS* co-segregates with SSRG60685K and BARCSOYSSR_18_1861. The genetic distance between BARCSOYSSR_18_1859 and BARCSOYSSR_18_1861 was reported to be 0.9 cM^34^. In our study, the genetic distance between these two SSR markers is 0.4 cM. The physical distance between BARCSOYSSR_18_1860 and BARCSOYSSR_18_1861 is 71 kb based on the soybean Williams 82 genome sequence (Fig. 5). The candidate annotated disease resistance gene-like sequence among the 10 predicted genes of the 92.7 kb *Rps13* region between SBP56.32 and BARCSOYSSR_18_1861 markers in the Williams 82 genome is an NB-ARC domain-containing gene, *Glyma.18g283200* (Table S5). There are three NB-LRR genes, *Glyma18g51930, Glyma18g51950,* and *Glyma18g51960*, identified from the *RpsJS*^34^ region between markers BARCSOYSSR_18_1859 and BARCSOYSSR_18_1861. The four NB-LRR genes with high similarity, are presumably paralogous sequences (Supplementary Fig. S1). They were identified from the Williams 82 haplotype that does not contain any known functional *Rps* genes. Based on the genetic and physical distances between BARCSOYSSR_18_1860 and BARCSOYSSR_18_1861 markers and differences in candidate NB-LRR-like resistance gene sequences, *Rps13* and *RpsJS* are unlikely allelic or the same gene.

We propose that the five *Rps* genes, *Rps4*, *6, 12, 13* and *JS,* might have evolved from a single progenitor *Rps* gene. Identification of these *Rps* genes will shed light on how *Rps* genes evolved in soybean to confer effector triggered immunity against a serious oomycete pathogen, *P. sojae*.

In this study we have shown that the broad-spectrum *Phytophthora* resistance is encoded by two *Rps* genes, *Rps12* and *Rps13,* with distinct race-specificity. The genetic distance between the two *Rps* genes is 4 cM. Therefore, to maintain the broad-spectrum *Phytophthora* resistance encoded by *Rps12* and *Rps13*, we must select both genes using molecular markers. We report here several SSR markers that should be ideal for introgressing *Rps12* and *Rps13* into new soybean cultivars.

## Supplementary Information

Supplementary Figure S1.

Supplementary Table S1.

Supplementary Table S2.

Supplementary Table S3.

Supplementary Table S4.

Supplementary Table S5.
