# Supplementary material for: Tightly linked Rps12 and Rps13 genes provide broad-spectrum Phytophthora resistance in soybean
Source: Sci Rep. 2021 Aug 19;11:16907. doi: 10.1038/s41598-021-96425-1 (PMC8377050; doi:10.1038/s41598-021-96425-1)
Supplement: Supplementary file 1 — Supplementary Figure S1. [file 41598_2021_96425_MOESM1_ESM.docx]

1. **(b)**

Glyma.18g283200 MADTIVVFLIEKLTRLLAEEAKLLGSAHDKVTSLRNELRFMNLFLNNSQGKRKDHNMVAE.


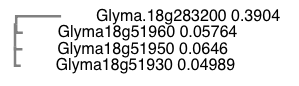
Glyma18g51960 MTDSVVAFVLDNLS-LLEDEHKLLSGVEDKVNSLCNELKFIHIFLKNSEGKR-SHDTGKE

Glyma18g51950 MADSVVVFLLDNLSRLLEDEHKLLSGVEDKINSLCNELKFIHIFLKNSEGKR-SHEMVKE

Glyma18g51930 MVDSVVTFLLDNLSRLLEDEHKLLSGVEDKINSLCNELKFIHIFLKNSEGKR-SHEMVKE

*.*::*.*::::*: ** :* ***... **:.** ***.*:::**:**:*** .*: *

Glyma.18g283200 LVDQIRDIAHEAEDVIDNYISDMIKQRRRNMLEKFGRGVDHALMLRNLTVKIDRIKTTIN

Glyma18g51960 VVSQIRDVAHKAENVVDTYVANIAQQKQRSKLSKLFHLKEHVMVLHQVNSEIEKIRSQIE

Glyma18g51950 VVSQIRDVTLKAEDVVDTYLSNIAQQKQRSKLSKLFHLKEHVMVLHQVNSDIEKIRTRID

Glyma18g51930 VVSQIRDVSLKAEDVVDTYLSNIAQQKQRSKLSKLFHLKEHVMVLHQVNSDIEKIRTRID

:*.****:: :**:*:*.*:::: :*..*. *.*: . :*.::*.::. .*:.*.: *:

**Supplemental Figure 1.** Alignment and phylogenetic relationships of the candidate NB-LRR genes of the *Rps13* and *RpsJS* regions.

Glyma.18g283200 is from the *Rps13* region.

Glyma18g51930, Glyma18g51950 and Glyma18g51960 are from the *RpsJS* region.

Glyma.18g283200 DIFDNKVKYGIEAGRRDSEE---EAERIRKQRRDVEEQEVVGFAHDSKVVIEKLMASGSR

Glyma18g51960 EIYKNGDRYGIGEGEFRSEEAAAEAESLLKRRREVEEEDIVGLVHDSSHVIHELMESESR

Glyma18g51950 EIYKNRDRYGIGEGDFRSEEAAAEAEPLLKRRREVEEEDVVGLVHDSSHVIQELMESESR

Glyma18g51930 EIYKNRDRYGIGEGDFRSEEAAAEAESLLKRRREVEEEDVVGLVHDSSHVIQELMESESR

:*:.* .*** * *** *** : *.**:***:::**:.***. ** :** * **

Glyma.18g283200 LKLVSIVGMGGLGKTTLARKIYNSNRVKNTFPCRAWGYASNDYRPREFFLSLLKC-LLST

Glyma18g51960 LKVVSIIGMGGLGKTTLARKIYNNNQVQLRFPCLAWVSVSNDYRPKECLLSLLKCSMSST

Glyma18g51950 LKVVSIIGMGGLGKTTLARKIYNNNQVQLWFPCLAWVSVSNDYRPKEFLLSLLKCSMSST

Glyma18g51930 LKVVSIIGMGGLGKTTLARKIYNNNQVQLRFPCLAWVSVSNDYRPKECLLSLLKCSMSST

**:***:****************.*.*: *** ** .******.* :****** : **

Glyma.18g283200 SKYNDLFKKREEASRSEEELKMKVRECLSRSGGKYLVVVDDVWQSQVWDEVKGAFPDDSN

Glyma18g51960 SEFEKL---------SEEDLKKKVAEWL--KGKSYLVVLDDIWETKVWDEVKGAFPDDQI

Glyma18g51950 SEFEEL---------SEEELKKKVAEWL--KGKKYLVVLDDIWETQVWDEVKGAFPDDQS

Glyma18g51930 SEFEKL---------SEEDLKKKVAEWL--KGKSYLVVLDDIWETQVWDEVKGAFPDDQI

*:::.* ***:** ** * * .* .****:**:*:::************.

Glyma.18g283200 GSRILITTRHAEVASHAGPMPPYFLPFLTEEESWELLSKKVFRGEDCPSDLEPMGKLIAE

Glyma18g51960 GSRILITSRNKEVAHYAGTASPYDLPILNEDESWELFTKKIFRGEECPSDLEPLGRSIVK

Glyma18g51950 GSRILITSRNKEVAHYAGTASPYYLPILNEDESWELFKKKIFGLEECPSDLEPLGRSIVK

Glyma18g51930 GSRILITSRNKEVAHYAGTASPYYLPILNEDESWELFTKKIFRGEECPSDLEPLGRSIVK

*******:*: *** :**. .** **:*.*:*****:.**:* *:*******:*. *.:

Glyma.18g283200 SCNGLPLAIIVMAGILANK-KSLRDWSRIKDHVNWHLGRDTT-LKDILKLSYDTLPARLK

Glyma18g51960 TCGGLPLAIVGLAGLVAKKEKSQREWSRIKE-VSWRLTQDKNGVMDMLNLRYDNLPERLM

Glyma18g51950 TCGGLPLAIVVLAGLVAKKEKSQREWSRIKK-VSWHLTEDKTGVMDILKLSYNNLPGRLK

Glyma18g51930 TCGGLPLAIVVLAGLVAKKEKSQREWSRIKE-VSWHLTEDKTGVMDILKLSYNNLPGRLK

:*.******: :**::*:* ** *:*****. *.*.* *.. : *:*:* *:.** **

Glyma.18g283200 PCFLYFGMYPEDYKIPVKQLIQLWISEGLLTQETCGSSTNIPEPEYIAEEYLDELVDRSL

Glyma18g51960 PCFLYFGICPRDY-----------------------------------------------

Glyma18g51950 PCFLYFGIYPEDYEISARQLIQYWIAEGFIQPQKTGIA-DTTELEDVADFYLDELVDRSL

Glyma18g51930 PCFLYFGIYPEDYEISARQLIQYWIAEGFIQPQKTGIA-DTTELEDVADFYLDELVDRSL

*******: * **

Glyma.18g283200 IQVVSRTSDGGVKTCRIHDLLRDLCISESKEDKFFEVCGEVD-FQIRDSCPLYKVKPNHW

Glyma18g51960 --------------------------VESYEER---------------------------

Glyma18g51950 VQVAKRRSDGGVKKCRIHDILRDLCLSESKSDKFLEVCTNSNIDTVSDTNP--RRMSIHW

Glyma18g51930 VQVAKRRSDGGVKTCRIHDLLRDLCLSESKYDKFLEVCTNSNIFTVSNTNP--RRMSFHW

** :.

Glyma.18g283200 R------------------------------WLLKSFRLARVLD---LGRMNVNSIPNDL

Glyma18g51960 ------------------------------------------------------------

Glyma18g51950 KPDSDVSANTFNKSCTRSMFIFGSDDRMDLDPVLKNFELARVLGCDMIQRVWSHTVSRDL

Glyma18g51930 KPDSDVSETTFNKSCTRSMFIFGRDAKTYLVPILKNFKLARVLGCDMIQQVWSYSASRDL

Glyma.18g283200 EKLIHLRYLRIHSYNIETIPASICRLWNLETLDLRGSPIKSFSGDLWQLKQLRHLLMFGP

Glyma18g51960 ------------------------------------------------------------

Glyma18g51950 KRMIHLRYLRI---EVEHLPDCVCSLWNLETLHVTYE--TTVSSKIWTLKRLRHLYLSGE

Glyma18g51930 KRMIHLRYLRI---EVEHLPDCVCSLWNLETLHVKYS--GTVSSKIWTLKRLRHLYLMGN

Glyma.18g283200 VGLPDMPSESKTMQNLQTLSTVALDPRT-TSLLDSRRFPRLTKLGIHHERRDKCNARIQL

Glyma18g51960 --------KAKELETVEVFIH---------------------------------------

Glyma18g51950 GKLPVVLPKTNRMENLQTLLLSGKYPQQIISLLNSGIFPRLGKLALRCPKTHAESA--ML

Glyma18g51930 GKLP--LPKANRMENLQTLVLSGDYPQQIIFLLNSGIFPRLRKLALRCYNSVEGPG--ML

::: ::.::.:

Glyma.18g283200 QSLNRLSHLRKLKVIGTTEIPQNANVFPSNITKISLTKFGCFNS--NAMHILGKLPSLQV

Glyma18g51960 ------------------------------------------------------------

Glyma18g51950 SSLHHLNNLHSLKVIEDLELPSDTNAFPSNLIKITLI-LAAFSNPHPLMKTLGRLTNLQI

Glyma18g51930 PSLQRLSNLHSLKVMRGCELLLDTNAFPSNLTKITLKDLHAFRDPQSLMKTLGRLPNLQI

Glyma.18g283200 LKLS-SQTNDTRFDLHCATGGFLQLQVFEMIAIKVKNWRLDKGSMPRIRRLDVRSCKSLT

Glyma18g51960 ------------------------------------------------------------

Glyma18g51950 LKLN-SGIDDILLDI--GSGEFPQLQLLHMRQIYVRQWRLEKDAMPRLRHLVIDNCYKLS

Glyma18g51930 LKVSFCMHNDIHLDI--GRGEFPQLQVLHMTQINVRQWRLEKDAMPRLRHLLIEECYGLS

Glyma.18g283200 ELPKELWSLTSLREVQVLWPCTELVKRLQNLVVNNGCKLVVYPLSTNDELDFL

Glyma18g51960 -----------------------------------------------------

Glyma18g51950 KLPEELWSLTALRVVHVLWPLEELANSLKDVEPRNGCKLIVSNASQLLE----

Glyma18g51930 ELPEELWSMTALRLVHVSWPSQELANSLKNVEPRNGCKLKIELLE--------
